# Protein glycosylation alterations in hepatocellular carcinoma: function and clinical implications

**DOI:** 10.1038/s41388-023-02702-w

**Published:** 2023-05-16

**Authors:** Yifei Wang, Huarong Chen

**Affiliations:** 1 School of Biomedical Sciences, The Chinese University of Hong Kong, Hong Kong, China; 2 Department of Anaesthesia and Intensive Care and Peter Hung Pain Research Institute, The Chinese University of Hong Kong, Hong Kong, China; 3 Institute of Digestive Disease and Department of Medicine and Therapeutics, State Key Laboratory of Digestive Disease, Li Ka Shing Institute of Health Sciences, CUHK Shenzhen Research Institute, The Chinese University of Hong Kong, Hong Kong, China

**Keywords:** Liver cancer, Tumour biomarkers

## Abstract

Hepatocellular carcinoma (HCC) is the third leading cause of cancer death worldwide. Understanding the cancer mechanisms provides novel diagnostic, prognostic, and therapeutic markers for the management of HCC disease. In addition to genomic and epigenomic regulation, post-translational modification exerts a profound influence on protein functions and plays a critical role in regulating various biological processes. Protein glycosylation is one of the most common and complex post-translational modifications of newly synthesized proteins and acts as an important regulatory mechanism that is implicated in fundamental molecular and cell biology processes. Recent studies in glycobiology suggest that aberrant protein glycosylation in hepatocytes contributes to the malignant transformation to HCC by modulating a wide range of pro-tumorigenic signaling pathways. The dysregulated protein glycosylation regulates cancer growth, metastasis, stemness, immune evasion, and therapy resistance, and is regarded as a hallmark of HCC. Changes in protein glycosylation could serve as potential diagnostic, prognostic, and therapeutic factors in HCC. In this review, we summarize the functional importance, molecular mechanism, and clinical application of protein glycosylation alterations in HCC.

## Introduction

Hepatocellular carcinoma (HCC), the primary malignancy of hepatocytes, is one of the leading causes of cancer death worldwide [1]. Despite the recent advance in the management of HCC, the majority of patients are diagnosed at advanced stages when tumors are ineligible for curative therapies such as surgical resection and liver transplantation, and the prognosis is very poor. Palliative approaches including transhepatic arterial chemotherapy and embolization (TACE) and targeted therapy are both recommended for treating advanced HCC, yet their survival benefits are modest. Thus, it is urgent to characterize the molecular mechanisms underlying the initiation and progression of HCC and identify potential molecular targets for diagnosis, prognosis, and treatment of HCC.

The development of HCC is a complex multistep process, involving the accumulation of genetic and epigenetic alterations. In addition, post-translational modifications (PTM) such as phosphorylation, glycosylation, acetylation, methylation, and ubiquitination are also involved in the process of cancer development through changing protein properties and functions. Glycosylation, one of the most common protein modifications, has attracted a lot of attention in the field of cancer biology over the past decades. Glycosylation refers to the process by which glycosidic linkages between saccharides and other saccharides, proteins, or lipids are formed. Glycosylation plays a key role in various cellular processes such as protein folding, modulation of receptor signaling, and control of immune recognition [2]. Recently, abnormal protein glycosylation alterations are considered a hallmark of cancer and are implicated in the malignant transformation of various cancer types including gastrointestinal, breast, lung, brain, ovarian, and hematologic cancers [3, 4]. Furthermore, dysregulated protein glycosylation has been reported to regulate tumor cell proliferation, metastasis, stemness, immune escape, and drug resistance [5]. Understanding the process of protein glycosylation and its contribution to HCC development will provide novel insights into the development of molecular biomarkers and therapeutic targets to improve the management of HCC. Here, we describe the change of protein glycosylation patterns in HCC, summarize its role and underlying molecular mechanisms in promoting HCC tumorigenesis and progression, and discuss the potential clinical implication of glycans for HCC diagnosis, prognosis, and treatment.

## Landscape of protein glycosylation

**Fig. 1:**
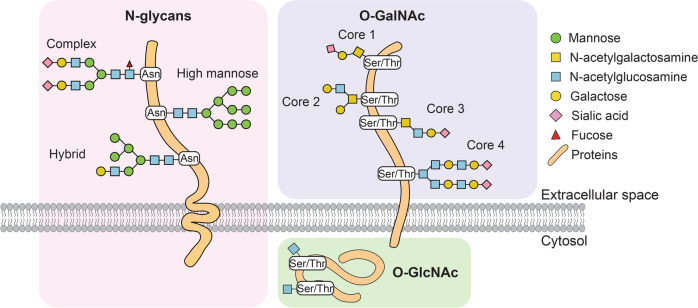
The structure of N-linked and O-linked glycans. N-linked and O-linked glycans are two common types of covalent modifications on proteins. They are regulated by a variety of enzymes and have strong effects on protein structure and function.

**Fig. 2:**
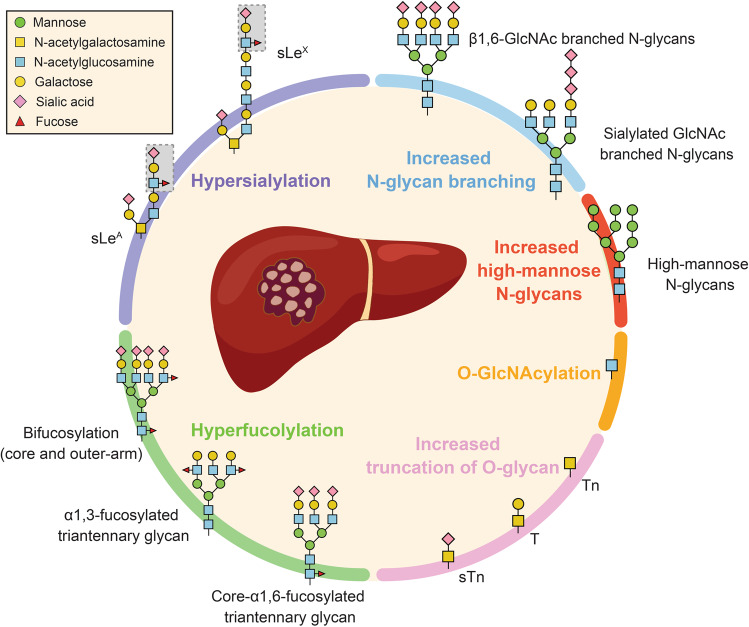
The change of protein glycosylation in HCC. HCC has been reported to be associated with dysregulation of several types of protein glycosylation, including core α-1,6 linked and α-1,3 linked outer-arm fucosylation, glycan branching, sialylation, fucosylation, and tetraantennary-linked glycan. These changes may affect the structure, stability, and function of glycosylated proteins in HCC cells and could potentially contribute to HCC development.

Glycoproteins can be identified in almost all living organisms. The term “glycome” describes all sugar chains (glycans) and glycoconjugates produced inside cells, the size of which could be 10–10^4^ times larger than the proteome across different species. Glycoconjugates are formed via the covalent linkage of glycans to lipid or protein molecules through a process called glycosylation. Glycosylation occurs in the endoplasmic reticulum (ER) and Golgi apparatus and is dynamically and intricately governed by regulatory machinery comprising various glycosyltransferases and glycosidases for glycan processing. The glycan composition, structure, and length are diverse, depending on the accessibility and activity of glycosylation enzymes, donor-acceptor substrates availability (e.g., UDP-galactose, UDP-N-acetylglucosamine, and GDP-fucose), the cell types and cellular signals. The N-linked and O-linked glycans (Fig. 1), the two most well-known glycoforms, are the oligosaccharides covalently added to a polypeptide backbone via N-linkage to Asparagine (Asn) and O-linkage to serine/threonine (Ser/Thr), respectively [2, 6]. Abnormal protein glycosylation alterations are now regarded as a hallmark of HCC. The change of protein glycosylation patterns is complex and dynamic, composing of many components which may interact with each other. In serum samples of HCC patients, several groups have reported a significant increase in the levels of core α-1,6 linked and α-1,3 linked outer-arm fucosylation, glycan branching, and sialylation [7–9]. As to HCC tissues, upregulated fucosylation and tetraantennary-linked glycan have been identified [10]. In the following parts, we will introduce altered O- and N-linked glycosylation, sialylation, and fucosylation in HCC (Fig. 2).

### O-linked glycosylation

O-Glycosylation is the addition of oligosaccharides to the oxygen atom of Ser/Thr residues in a protein. O-N-acetylgalactosamine (O-GalNAc) and O-N-acetylglucosamine (O-GlcNAc) are two common types of O-glycosylation [11]. O-GalNAc usually occurs on the secreted proteins after the protein has been folded and transported to the Golgi apparatus [12]. In contrast, O-GlcNAc is found on intracellular proteins in the nucleus, cytoplasm, and mitochondria. The GalNAc transferase enzyme is responsible for the addition of GalNAc group to the proteins [13]. Different O-GalNAc glycans from different sites would lead to diverse molecular functions of proteins. Similar to O-GalNAc, O-GlcNAc is a dynamic and reversible process controlled by O-GlcNAc transferases (OGTs) and O-GlcNAcases (OGAs) which catalyze the attachment and removal of GlcNAc group, respectively [14]. O-GlcNAc could exert distinct roles as determined by their subcellular localizations in cells. Cytosolic O-GlcNAc is regarded as an important regulatory modification to signal transduction, while nuclear O-GlcNAc can modulate the activity of transcriptional regulators. Notably, O-GlcNAc would crosstalk with phosphorylation in several ways, e.g., through competition for the same Ser/Thr residues, or influencing each other nearby, thus adding the functional complexity of proteins [15].

### N-linked glycosylation

N-Glycosylation is the addition of oligosaccharides to the nitrogen atom of the Asn residue of the Asn-X-Ser/Thr sequence in glycoproteins, where X could be any amino acid except proline (Pro). All eukaryotic N-glycans are synthesized in a similar way and share a common core sequence, Manα1-3(Manα1-6)Manβ1-4GlcNAcβ1–4GlcNAcβ1–Asn-X-Ser/Thr. Determined by the protein and cell types, N-glycans are classified into three subtypes: high-mannose (addition of mannose residues), complex oligosaccharides (addition of GlcNAc residues), and hybrid oligosaccharides (addition of a mannose on one side of the branch, and GlcNAc on the other side which initiates a complex branch) [16]. N-linked glycans exert various functions such as modulating protein stability and solubility, directing the trafficking of protein, and mediating cell signaling.

### Sialylation

Sialylation is the attachment of sialic acid to the terminal position of glycan chains on glycoconjugates. Sialylation plays a crucial role in the post-translational modification of proteins and is implicated in embryonic development, neurodevelopment, and immune responses. Sialic acid, a class of alpha-keto acid sugars with a nine-carbon backbone, is widely distributed in animal tissues. Over 50 kinds of sialic acid are identified, which play critical roles in modulating various interactions, such as cell-matrix and cell-cell interactions. The process of sialylation is controlled by sialyltransferases and sialidases, which respectively conjugate and cleave sialic acid residues. Sialyltransferases are a family of glycosyltransferases which are expressed in the Golgi apparatus and function in a tissue- and substrate-specific manner [17]. Different sialyltransferases could catalyze the formation of different glycosidic linkages, such as α-2,3, α-2,6, or α-2,8 linkages.

### Fucosylation

Fucosylation refers to the transfer process of fucose sugar units from GDP-fucose to N-glycans, O-glycans, glycolipids, and glycoproteins, a process that ubiquitously exists in mammals [18]. Generally, fucosylation is regarded as the terminal modification of glycan structures and can be classified into core- and outer-arm (terminal) types based on the fucose location [19]. Fucosyltransferases (FUTs) are the major enzymes that catalyze fucosylation. There are thirteen FUTs discovered in the human genome, of which FUTs in the Golgi apparatus initiate N-linked fucosylation while ER-localized O-fucosyltransferases (protein O-fucosyltransferase 1–2 [PoFucT1-2]) induce O-linked fucosylation [20]. The substrate specificities of distinct FUTs are essential for multiple physiological and pathological processes.

## Protein glycosylation alterations in HCC

A wide range of glycosylation aberrations is identified in HCC compared to the non-tumoral counterparts including O-GalNAcylation, O-GlcNAcylation, fucosylation, and sialylation [21]. Accumulating evidence demonstrates that these alterations would modulate pro-tumorigenic transcription, signal transduction, cell-cell interaction, and cell-matrix adhesion, and ultimately contribute to tumor initiation and progression. Here, we discuss the function of distinct glycosylation patterns in regulating HCC proliferation, migration, invasion, stemness, and tumor immune tolerance.

### O-GalNAc and O-GlcNAc in HCC

Aberrant O-GalNAcylation and O-GlcNAcylation are key features of HCC. GALNTs-encoded GalNAc-T glycosyltransferases are primary enzymes in the initial step of mucin-type O-glycosylation and are responsible for liver malignant transformation. Dysregulation of GALNT family members is commonly observed in HCC [22]. miR-9 could directly interact with GALNT4 to repress its expression, while Hnf4α elevated GALNT10 levels through the downregulation of miR-122 in hepatitis B virus (HBV)-infected hepatoma cells [23]. Different GALNTs might exert opposite functions in HCC. GALNT2 and GALNT4 were found to promote the O-glycan modifications of EGFR to suppress EGF-potentiated HCC pathogenesis [24, 25]; on the contrary, GALNT1 and GALNT10 contributed to HCC malignancy via inducing O-glycosylation-mediated EGFR pathway activation [23, 26]. These discordant findings suggest that GALNT family members may have distinct preferences for GalNAc-modification sites on EGFR to cause diverse protein functions. Of note, GALNTs could transport from the Golgi apparatus to ER for activation in human and mouse liver cancer [22]. As such, mice expressing ER-targeted GALNT1 (ER-G1) were prone to develop early-stage tumors with rapid tumor expansion and reduced survival [22]. Further mechanistic investigations identified that ER-G1 promoted extracellular matrix (ECM) degradation via glycosylating the MMP14, leading to accelerated tumor growth [22]. In addition to GALNTs, core 1 β1,3-galactosyltransferase (C1GALT1), the primary enzyme that controls the Tn antigen biosynthesis, is also responsible for hepatocarcinogenesis [27, 28]. Overexpression of C1GALT1 in HCC stimulated hepatocyte growth factor (HGF) signaling by modulating the binding of MET with two O-glycans (Vicia villosa agglutinin and peanut agglutinin) and its dimerization, leading to elevated cell proliferation; conversely, blockade of MET using PHA665752 attenuated C1GALT1-driven HCC development [27]. Consistently, overexpression of C1GALT1 potentiated HCC metastasis by decorating integrin β1 with O-glycans [28].

In addition to O-GalNAc, hyper-O-GlcNAcylation is also prevalent in HCC, which has been reported to facilitate the malignant transformation of hepatocytes and promote HCC proliferation, metastasis, and stemness [29]. Several factors, such as HBV infection and high glucose, are known to contribute to increased O-GlcNAcylation in HCC [30]. In human HCC cell lines and tumor tissues, higher levels of O-GlcNAc modifications are observed [31]. A positive feedback loop between O-GlcNAcylation and Sine oculis homeobox homolog 1 (SIX1) expression has been identified in HCC [31]. On one hand, SIX1 elevated the level of O-GlcNAcylation inside HCC cells; on the other hand, O-GlcNAcylation of SIX1 at Thr276 prevented SIX1 from ubiquitination-mediated degradation, thus promoting HCC proliferation [31]. O-GlcNAcylation of Speckle-type POZ protein (SPOP) at Ser96 led to the translocation of SPOP from cytoplasm into nucleus, thus attenuating the ubiquitination of the oncoprotein neurite outgrowth inhibitor-B (Nogo-B) and promoting HCC progression [32]. SLC35B4 acted as an essential transporter of UDP-GlcNAc to stabilize c-Myc via modifying its O-GlcNAcylation. Subsequently, elevated c-Myc expression in HCC cells enhanced cell proliferation and migration [33]. Rab3A was identified as a metastatic suppressor in HCC; however, O-GlcNAcylation of Rab3A inactivated Rab3A by regulating its GTP-binding activity, leading to reduced mitochondria oxidative phosphorylation (mtOXPHOS) in HCC [34]. Notably, the link between O-GlcNAcylation and RNA N6-methyladenosine (m6A) has been reported to facilitate HBV-associated HCC tumorigenesis [30]. OGT catalyzed O-GlcNAcylation of YTHDF2, an m6A binding protein, at Ser 263 to prevent ubiquitination-mediated degradation of YTHDF2 protein in HCC; subsequently, upregulated YTHDF2 promoted HCC proliferation via enhancing mRNA stability of m6A-modified MCM2 and MCM5 [30]. Inhibition of OGT by OSMI-1 effectively hampered HBV-related hepatocarcinogenesis in vivo [30]. In addition to HCC proliferation and migration, O-GlcNAc modification also affects HCC stemness. OGT-induced O-GlcNAcylation of eIF4E endowed HCC cells with stem-like features as exemplified by a higher proportion of CD133^+^ cells, possibly through increasing the physical interaction between eIF4E and 5’UTR of SOX2 [35].

High blood sugar (hyperglycemia) is known to promote HCC development. However, the underlying molecular mechanisms remain unknown. Numerous studies have demonstrated that high glucose could stimulate the hexosamine biosynthesis pathway (HBP)-mediated synthesis of UDP-GlcNAc, the substrate of OGT, to elevate cellular O-GlcNAcylation. Thus, hyper O-GlcNAcylation might be implicated in high-glucose-associated HCC. Buren S et al. reported a functional complex of co-chaperone URI, PP1γ, and OGT, which was maintained by glucose [36]. In response to glucose deprivation, URI was phosphorylated by PKA at Ser-371, leading to the release of PP1γ and URI-mediated OGT inhibition. Consequently, O-GlcNAcylation was reduced inside cells to promote c-MYC degradation to maintain cell survival. Conversely, the presence of glucose increased OGT and c-MYC levels, thereby accelerating liver tumorigenesis [36]. In addition, high glucose could potentiate liver tumorigenesis by stimulating the O-GlcNAcylation of Yes-associated protein (YAP) at Thr241 [37]. O-GlcNAcylation of YAP enhanced the expression, stability, and function of YAP, which in turn activated the transcription of potent oncogenic factors and HBP-related genes (e.g., OGT, Nudt9, and SLC5A3), which mediate O-GlcNAcylation. Thus, the positive feedback between YAP and O-GlcNAcylation is essential for high-glucose-induced liver tumorigenesis [37]. A consistent observation is identified between O-GlcNAcylation and β-catenin, which cooperatively facilitate HCC growth [38]. In this study, upregulation of O-GlcNAc induced by high glucose promoted β-catenin expression, which in turn increased the HBP pathway and O-GlcNAc level probably through elevating UAP1 [38].

### N-glycosylation in HCC

Aberrant change of N-glycans is another key factor that influences the malignant properties of HCC. Alpha-1,3-Mannosyl-Glycoprotein 2-Beta-N-Acetylglucosaminyltransferase, encoded by MGAT1 gene (also termed as GNT-I), is required to converse high-mannose to hybrid and complex N-glycans. MGAT1 is found to be closely associated with the dedifferentiation of HCC [39]. N-glycosylated Mer Tyrosine Kinase (MERTK) is indispensable for tumor growth [40]. Mechanistic investigations have revealed that N-glycosylation of MerTK at Asn294 and Asn454 increased MerTK protein expression by protecting it from ubiquitin-dependent degradation, which in turn activated the Warburg effect and Akt/GSK3β signaling to enhance HCC growth [40]. Aberrant change of N-glycans also regulates HCC metastasis. β1,6-GlcNAc-branched glycan of CD147/basigin by *N*-acetylglucosaminyltransferase V (GnT-V) upregulated matrix metalloproteinases (MMPs) expression (e.g., MMP-1, MMP-2, and MMP-9) and enhanced their associations with integrin β1, thereby contributing to HCC metastasis [41]. In addition, N-acetylglucosaminyltransferase IVa (GnT-IVa) could upregulate core-α-1,6-fucosylated triantennary glycan (NA3Fb), a specific N-glycan on the surface of malignant hepatocytes, to facilitate HCC cell migration and invasiveness [42]. Removal of N-glycans may also contribute to HCC progression. Blockade of N-glycosylation of GP73, a type II Golgi transmembrane protein, at Asn109 or Asn144 was found to promote HCC mobility; and deletion of Asn144 inhibited cell adhesion [43]. N-glycan alteration also influences the host immune response. In response to IL6, JAK1 phosphorylated PD-L1 at Tyr112 to catalyze PD-L1 glycosylation which was mediated by ER-associated N-glycosyltransferase STT3A, leading to elevated PD-L1 stability [44]. In contrast, the administration of ruxolitinib, a selective JAK1/2 inhibitor, destabilized PD-L1 and improved the therapeutic efficacy of anti-Tim-3 immune checkpoint therapy in HCC [44].

### Sialylation in HCC

Sialylated glycans play an essential role in cancer by regulating cell adhesion, pro-tumorigenic signaling, and immune response. In the 1970s, gradually upregulated α2,6-sialylation levels were observed in the serum and tumoral tissue of transgenic mice, which developed HCC [45]. Since then, mounting research has been conducted, indicating that increased sialylation, especially α2,6- and α2,3-sialylation, contributes to the development of HCC. N-glycolylneuraminic acid (Neu5Gc), a nonhuman sialic acid that is unable to be generated by humans because of a lack of CMP-sialic acid hydroxylase (CMAH), is highly enriched in red meat. Samraj et al. reported that dietary Neu5Gc plus administration of anti-Neu5Gc antibodies led to a significantly higher incidence of HCC in *Cmah*^*−/−*^ mice which developed systemic inflammation [46]. High incorporation of food-derived Neu5Gc was identified in the tumors, implying that hepatic incorporation of Neu5Gc could exacerbate tumor formation [46]. A range of sialyltransferases (STs) and sialidases (NEUs) are dysregulated in HCC, leading to increased sialylation, which is associated with cancer progression [47, 48]. Caveolin-1 was reported to stimulate ST6GAL1 transcription to elevate α2,6-linked sialic acid at the cell surface [49]. Moreover, microRNA and lncRNA also regulate sialylation in HCC. Long noncoding RNA TINCR acted as a competing endogenous (ceRNA) to sponge miR-195-3p, thereby protecting ST6GAL1 from miR-195-3p-induced repression; subsequently, elevated ST6GAL1 promoted HCC progression and chemoresistance [50]. In addition, miR-26a, miR-548l, and miR-34a have been reported to inhibit the expression of ST3GAL5, which encodes Lactosylceramide alpha-2,3-sialyltransferase, to suppress HCC cell proliferation and migration [51]. miR-26a also negatively regulated ST3GAL6, a Type 2 lactosamine alpha-2,3-sialyltransferase, leading to the inhibition of Akt/mTOR signaling and reduced HCC growth both in vitro and in vivo [52].

Abnormal sialylation plays a predominant role in regulating HCC metastasis. The negatively charged sialic acid empowers tumor cells with anti-adhesive features that enhance tumor mobility, migration, and invasiveness. In mouse hepatocarcinoma H22 cells, an increase of α2,6-sialylated α5-subunit in cell surface was found to potentiate cell adhesion to fibronectin [49]. ST6GAL1-induced α2,6-sialylation is also implicated in exosome-mediated proliferation and migration [53, 54]. Knockdown of ST6GAL1 significantly reduced the α2,6-sialylation level of CD63 on the surface of HCC-derived exosomes, leading to inactivated Akt/GSK-3β and JNK1/2 signaling pathways, which ultimately led to impaired HCC proliferation and metastasis [53]. Additionally, loss of ST6GAL1 inhibited the α2,6-sialylation of neutral sphingomyelinase-2 (nSmase2) and the nSmase2-dependent sorting of miRNA into exosome, especially miR-100-5p [54]. Exosome-derived miR‑100‑5p increased the migrative and invasive capabilities of recipient HCC cells [54]. Besides ST6GAL1, other sialyltransferases such as ST3GAL1 are also important for HCC growth and metastasis. ST3GAL1-deficient HCC cells exhibited impaired cell proliferation, migration, and invasiveness [47]. However, dysregulated sialylation has also been reported to play a tumor-suppressive role in HCC. Zou X et al. revealed that human HCC cell lines expressing high ST6GAL1 displayed lower metastatic capability [48]. Mechanistic investigations showed that ST6GAL1-induced upregulation of α2,6-sialylation on melanoma cell adhesion molecule (MCAM) disrupted the interaction between MCAM and galectin-3, leading to impaired MCAM dimerization and consequent suppression of HCC metastasis both in vitro and in vivo [48]. These discrepant observations might be explained by the opposite tendency between α2,6 sialylation and fucosylation, given that blockade of fucosylation could elevate α2,6 sialylation in HepG2 cells in a dose-dependent manner due to the competitive binding of fucosyltransferases and sialyltransferases to the same substrate [55, 56]. Therefore, sialylation and fucosylation could coordinately contribute to hepatic malignancy.

In contrast with the role of STs, NEUs remove sialic acid from glycoconjugates. NEU4 has been reported downregulated in HCC tissues, and low NEU4 expression is correlated with high grade and poor outcomes of HCC [57]. Mechanistically, NEU4 can remove α-2,3-sialic acids from the end of the oligosaccharide chains on the CD44, resulting in enhanced cell-matrix adhesion and consequent suppression of HCC metastasis both in vitro and in vivo [57].

### Fucosylation in HCC

Hyperfucosylation of glycoproteins is also closely related to HCC development. In situ imaging of N-linked glycans revealed an increased abundance of fucosylated N-linked glycans in HCC tissues compared to cirrhotic and adjacent normal tissues [58]. Moreover, high fucosylated glycoform was associated with poor survival of HCC patients [58]. Elevated expression of regulatory factors such as fucosyltransferases (FUT), increase in GDP-fucose abundance, and high GDP-fucose transport activity collaboratively contribute to hyperfucosylation [59, 60]. Among them, α1,6-fucosyltransferase (FUT8) controls core fucosylation. A stepwise increase of FUT8 expression in the serum and liver have been reported during the development of HCC [61]. Caveolin-1 increased FUT8 expression via activating Wnt/β-catenin pathway, thereby facilitating the proliferation and migration of mouse HCC cell lines [62]. Besides, elevated FUT8 expression could stem from the HOTAIR/P300/STAT3 cascade-dependent transcriptional activation [63]. HBV-encoded X protein (HBx) is known to contribute to the pathogenesis of viral-induced HCC. Notably, HBx could upregulate FUT2 and FUT2-induced Globo H expression by disrupting the binding of miR-15b to the 3’-UTR region of FUT2, resulting in increased HCC growth both in vitro and in vivo [64]. Similarly, Hepatitis C virus (HCV) infection promoted the expression of FUT8 and subsequent fucosylated ANXA2 [65]. HCV-induced FUT8 expression not only facilitated HCC cell proliferation via activating PI3K-AKT-NF-κB but also conferred resistance to 5-fluorouracil (5-FU) [66]. Ectopic FX expression promoted GDP-L-fucose biosynthesis and subsequent core-fucosylation of α-fetoprotein (AFP), a Food and Drug Administration (FDA)-approved biomarker for early HCC diagnosis [59]. Moreover, SP1 transactivated GDP-fucose transporter 1 (SLC35C1) to increase the fucosylation of haptoglobin (Hp), whereas inhibition of SP1 using mithramycin A decreased fucosylated Hp expression in HepG2 cells [67].

FUT8-directed core fucosylation plays an important role in driving HCC [61, 63]. Loss of FUT8 downregulated cell-cycle related genes to suppress the growth of HCC xenografts and inhibit HCC formation of Fut8-specific knockout mice (*Fut8*^*−/−*^) treated with Diethylnitrosamine (DEN) plus pentobarbital (PB) [61]. In this study, FUT8 deficiency attenuated the phosphorylation of Akt, EGFR, c-MET, and ERK in HCC cells upon EGF and HGF stimulation [61]. Moreover, FUT8 was reported to enhance the core fucosylation of Hsp90 [63]. FUT8-catalyzed α1, 6-fucosylated Hsp90 strengthened the interaction between MUC1 and p-STAT3, which subsequently activated JAK1/STAT3 to promote HCC [63]. Intriguingly, activated STAT3, in turn, bound to the promoters of FUT8 and MUC1 directly to induce their transcription, thus establishing a positive feedback loop between FUT8 and STAT3 in HCC [63]. In addition to modulation of cell proliferation, FUT1-directed fucosylation also regulates the plasticity of HCC. High FUT1 expression was closely associated with HCC aggressiveness; functionally, ectopic FUT1 expression promoted the growth and self-renewal of HCC cells, while inhibition of FUT1 exerted the opposite effects [68]. Upon glucose deprivation, PERK/eIF2α/ATF4 axis induced the transcription of FUT1 which in turn activated AKT/mTOR/4EBP1 pathway through targeting glycoproteins such as including CD147, ICAM-1, EGFR, and EPHA2, resulting in enhanced stem-like property of HCC [68].

## Clinical implications of altered glycosylation in HCC

Aberrant glycosylation frequently occurs in HCC and contributes to the malignant phenotypes, implying the clinical potential of glycans for HCC management. With the advancement of glycomics technology, several cancer glycobiomarkers have been uncovered that present diagnostic and/or prognostic values in HCC. Glycan-based treatments, e.g., inhibition of glycosylation, have also shown some potential to suppress HCC. Moreover, the detection of specific glycomic signatures could be used to predict the response to anti-cancer therapy. Here we summarize the potential clinical implications of altered glycosylation in HCC (Table 1).

**Table 1:** Serological glycome biomarkers for HCC diagnosis.

Marker	Glycoprotein or glycoform	Expression	Technique	Clinical relevance	Performance	Ref
AFP-L3	Fucosylation	Increase	Lectin affinity electrophoresis	Diagnosis & early detection	–	[92]
NA3Fb	Fucosylation	Increase	DSA-FACE	Discriminate HBV-related HCC from LC	AUC = 0.78–0.84	[93]
		Increase	DSA-FACE	Discriminate HBV-related HCC from fibrosis	AUC = 0.851	[94]
Log (NG1A2F/NA3Fb)	N-glycan	Decrease	DSA-FACE	Discriminate HBV-related HCC from fibrosis	AUC = 0.873	[94]
Hemopexin glycan marker[(NA3Fcb NA4Fb)/NA2]	N-glycan	Increase	DSA-FACE	Diagnose HCC	AUC = 0.92	[71]
				Discriminate cirrhotic HCC from LC	AUC = 0.82	
The combination of glycan 1, glycan 5, and glycan 6	N-glycan	Increase in glycan 5; Decrease in glycan 1 and glycan 6	MALDI-TOF MS	Discriminate HCV-related HCC from CLD	AUC = 0.96	[8]
The combination of complement C3, ceruloplasmin, histidine-rich glycoprotein, CD14, and hepatocyte growth factor	Fucosylation	Increase	Lectin-antibody arrays	Diagnose early-stage HCC	AUC = 0.811	[95]
Paraoxonase 1(PON1)	Fucosylation and sialylation	Increase	Tandem lectin affinity chromatography	Discriminate HBV-related HCC from LC	AAL-reactive fraction: AUC = 0.892; WGA-reactive fraction: AUC: = 0.902	[96]
	Fucosylation	Increase	Lectin ELISA	Discriminate HBV-related HCC from LC	AUC = 0.803	[97]
Haptoglobin (Hp)	bifucosylation	Increase	MALDI-QIT-TOF MS	Discriminate ALC-related HCC from ALC-related LC	For ALC-related HCC:AUC = 0.843	[73]
				Discriminate HCV-related HCC from LC	For HCV-related HCC:AUC = 0.821	
Ceruloplasmin (CERU)	Site-specific fucosylation	Increase	Nano LC-LTQ-MS	Diagnose ALC-related HCC	AUC = 0.838–0.922	[77]
α-1-antitrypsin(A1AT)	Fucosylation	Increase	Lectin FLISA	Diagnose HCC	AUC = 0.75	[79]
	Core fucosylation	Increase	MALDI-TOF MS;Lectin FLISA	Diagnose HCC	AUC = 0.871	[78]
kininogen	Core fucosylation	Increase	Lectin FLISA	Diagnose HCC	AUC = 0.79	[79]
GP73	Fucosylation	Increase	Lectin ELISA	Diagnose HCC	AUC = 0.885	[98, 99]
hemopexin	Fucosylation	Increase	Lectin FLISA	Diagnose HCC	AUC = 0.9512	[100]
fetuin-A	Fucosylation	Increase	Lectin FLISA	Identify HCC from non-HCC	AUC = 0.8691	[100]
A2G1(6)FB	N-glycan	Increase	HPLC	Discriminate HCV-related HCC from LC	AUC = 0.9641	[101]
13 N-glycans based LR algorithm	N-glycan	Increase	N-glycan fingerprint	Identify AFP-negative HCC	Training cohort: AUC = 0.842 (0.784–0.899); Validation cohort:AUC = 0.860 (0.824–0.897)	[70]

*ALC* alcohol-related HCC, *AUC* area under the receiver operating characteristic (ROC) curve, *CLD* chronic liver disease, *DSA-FACE* DNA sequencer-assisted fluorophore-assisted carbohydrate electrophoresis, *HBV* hepatitis B virus, *HCC* hepatocellular carcinoma, *HPLC* high-performance liquid chromatography, *LC* liver cirrhosis, *LC-LTQ-MS* liquid chromatography with linear ion trap mass spectrometry, *MALDI-QIT-TOF MS* matrix-assisted laser desorption/ionization quadrupole ion trap time-of-flight mass spectrometry, *MALDI-TOF MS* matrix-assisted laser desorption/ionization time-of-flight mass spectrometry.

### Altered glycosylation and HCC diagnosis

Serum AFP is the most widely used biomarker to screen and diagnose HCC, however, the performance is suboptimal. Up to 40–50% of HCC patients do not exhibit elevated AFP levels, raising the importance of identifying new biomarkers and determining their significance. Distinct glycosylation patterns have been reported between low- and high-AFP HCCs. Through integrated glycoproteomic and proteomic analysis, Zhao T et al. uncovered several sialylated but not core fucosylated triantennary glycans that were significantly increased in HCC patients with low AFP levels compared to those high-AFP-expressing HCCs and normal subjects [69]. Besides, Huang C et al. examined serum N-glycan structures abundances in 1340 participants including AFP-negative HCC, chronic liver diseases, and healthy controls using N-glycan fingerprint technology, and 13N-glycan was selected as the most significant structure for distinguishing AFP-negative HCC and non-HCC patients by Lasso algorithm [70]. In this study, they established a biomarker panel of 13N-glycan structures using logistic regression (LR) model which exhibited a high diagnostic accuracy of HCC for AFP negative subjects in both training (Area under the ROC Curve (AUC) = 0.842) and validation (AUC = 0.860) cohorts [70]. Consistently, Goldman R et al. revealed an altered abundance of 57 N-glycans in the serum of HCC patients compared with controls [8]. Furthermore, they stated that the combination of three identified N-glycans could achieve 90% prediction accuracy to classify HCC in a population with HCV infection prevalence [8]. In a Belgian cohort, the detection of branching α-1,3-fucosylated multiantennary glycans on hemopexin could diagnose HCC patients with cirrhosis with an AUC of 0.92 which was superior to that of AFP (AUC = 0.82) [71]. Shang S et al. developed a magnetic beads-based lectin ELISA system to measure serum fucosylated Hp in a separate cohort of 260 subjects comprising 130 HCC patients and 130 healthy donors [72]. They found that quantification of Hp fucosylation level had a good diagnostic performance for HCCs regardless of AFP expression [72]. In line with this, bifucosylated Hp was capable of diagnosing HCV-associated HCC with an AUC of 0.821 [73]. All these findings highlight the clinical relevance of glycan-based biomarkers for the diagnosis of HCC.

Detection of the glycan-based biomarker in the serum is not only a promising non-invasive approach to distinguish HCC patients from healthy individuals but also greatly improves the diagnostic performance of AFP. AFP-L3, an N-glycosylated isoform of AFP, can be detected only in HCC but other liver diseases. AFP-L3 has been approved by FDA as an HCC biomarker that could distinguish HCC from liver cirrhosis (LC) with high accuracy [74–76]. Detection of the core-fucosylated site (site 138) in ceruloplasmin (CERU) can be used to predict alcohol-related HCC, and adding AFP would further increase the diagnostic accuracy (AUC = 0.945) [77]. Moreover, several studies have reported that fucosylation of α-1-antitrypsin (A1AT), in particular core α-1,6 fucosylated A1AT (Fc-A1AT), could be used independently to distinguish between cirrhosis and HCC [78, 79]. Fucosylated kininogen (Fc-Kin) also achieves good performance for the diagnosis of HCC of different stages with an AUC of 0.79, and adding AFP and GP73 would further increase the diagnostic accuracy (AUC = 0.94) [79].

### Altered glycosylation and HCC prognosis

Glycan-based biomarkers are also useful to predict the outcome of HCC patients. Huang C et al. developed an LR algorithm named Car-G based on serum 13N-glycan structure abundance to assess the risk score of patients with AFP-negative HCC; they identified that patients with high Car-G score exhibited poor post-operative overall survival (OS)and relapse-free survival (RFS) [70]. By quantifying N-glycosylated modifications of IgG molecules derived from tumor tissues of 151 HCC patients undergoing surgical resection, Wu RQ et al. reported that increased sialylated IgG abundance could serve as an independent prognostic factor of favorable disease-free survival (DFS) [80]. In a Japanese cohort of 369 HCC patients undergoing primary curative hepatectomy, Kamiyama T et al. demonstrated that the detection of sialylated N-glycans G3560 and G2890 in the serum could respectively predict survival and post-operative recurrence of HCC patients [81].

Alternatively, regulators of glycosylation processes could also serve as potential prognostic biomarkers in HCC. Tang H et al. developed a glycoscore system based on the relative expression of glycosylation-regulating signature, through which HCC patients were divided into low- and high-glycoscore patterns [82]. They found that HCC patients with high-glycoscore had a significantly shorter OS than those with low-glycoscore [82]. FUT1 adds a fucose through an α1,2-linkage to the terminal galactose of glycoconjugates. High FUT1 expression has been reported as an independent poor prognostic factor for patients with HCC [68, 83]. ST6GAL1 transfers sialic acid from CMP-sialic acid to galactose-containing substrates. Lower ST6GAL1 expression was found to correlate with shorter OS and RFS in HCC [84]. OGA catalyzes the removal of O-GlcNAc on serine and threonine residues of proteins. In a cohort of sixty HCC patients undergoing liver transplantation, lower OGA mRNA expression in tumor specimens was associated with worse postoperative recurrence-free survival (RFS) [29].

### Altered glycosylation and HCC therapy

Dysregulated glycosylation has been reported to be associated with therapy resistance of HCC. In epirubicin- and mitoxantrone-resistant HCC cells, Kudo T et al. observed elevated core-fucosylated triantennary oligosaccharides compared to their parental cells, concomitant with altered mRNA expressions of glycosyltransferases synthesizing such as GnT-IVa, GnT-IVb and FUT8 [85]. In support of this, alterations of FUTs are thought to be involved in the tumor multidrug resistance (MDR) of HCC. Different fucosylated N-glycans profiles were revealed between 5-FU-resistant BEL7402 (BEL/FU) cells and the parental BEL7402 by mass spectrometry (MS) analysis [86]. Among the FUT family, FUT4, FUT6, and FUT8 were highly expressed in MDR HCC cell lines which could influence the therapy response of HCC by regulating PI3K/Akt signaling and MDR-related protein 1 (MRP1) [86]. Consistently, overexpressed FUT8 in HCV-infected HCC induced the expression of drug-resistant proteins such as P-glycoprotein (P-gp) and MRP1 to render 5-FU resistance of HCC cells; targeting of FUT8 or P-gp/MRP1 restored the sensitivity of HCC cells towards 5-FU treatment [66]. Sorafenib is a multikinase inhibitor approved by FDA for treatment of advanced HCC. The administration of sorafenib to HCC cells was found to alter protein glycosylation as exemplified by increased α-1,3GalNAc/Gal, β-1,3 Gal, GalNAcα-Ser/Thr(Tn) and α-GalNAc structures while decreased GlcNAc, sialic acid, tetra-antennary complex-type N-glycan and β-1,4 Gal structures [87]. Thus, sorafenib may suppress HCC cells by changing chain structures of glycoproteins. Meanwhile, the changes in protein glycosylation of HCC cells would increase the resistance to sorafenib. Upregulation of FUT1 has been reported to confer HCC cells with sorafenib resistance, and pharmacological inhibition of α1,2-fucosylation by 2-deoxy-D-galactose (2DGal) enhanced the therapeutic efficacy of sorafenib [68]. GALNT10 is responsible for the initial reaction of O-linked (mucin-type) oligosaccharide biosynthesis. GALNT10-deficient cells were more sensitive to both sorafenib and doxorubicin treatment as evidenced by significantly elevated cell apoptosis, suggesting the potential of GALNT10 as a therapeutic target for HCC [23]. Hypersialylation is also correlated with drug resistance in cancer. Sialyltransferase ST6GAL1 increased oxaliplatin resistance by activating NF-κB signaling in HCC [50]. Meanwhile, ST6GAL1 could also modulate p38 MAPK/caspase-dependent pathway to protect HCC cells from docetaxel-induced cell apoptosis [88]. Notably, among HCC patients with high serum ST6GAL1 levels who received tyrosine kinase inhibitors (TKI) therapy, lenvatinib conferred better survival than sorafenib [89], implying that detection of serum ST6GAL1 is useful in guiding the selection of appropriate drug therapy for HCC patients.

Apart from chemo- and targeted therapies, aberrant glycosylation also impacts the efficacy of immunotherapy. Suppression of IL6/JAK1-mediated PD-L1 glycosylation sensitized anti-Tim3 immune checkpoint blockade (ICB) therapy as manifested by reduced tumor growth and prolonged survival in Hepa1-6 hepatoma-bearing mice [44]. In this study, a combination of anti-IL-6 and anti-Tim-3 treatments produced a synergistic effect to enhance cytotoxic CD8^+^ T activity in the tumor microenvironment [44]. Effector T cells, including cytotoxic CD8^+^ T, play a pivotal role in the anti-tumor immune response. Upon anti-PD1 administration, effector T cells elevated the sialylation of IgG in an IFN-γ-ST6Gal-I-dependent mechanism [80]. Sialylated IgG then bound to macrophages expressing type II Fc receptors DC-SIGN to stimulate ATF3 expression which in turn deactivated cGAS-STING pathway and abrogated the antitumorigenic immunity of type I IFN in HCC [80]. Inhibition of IgG sialylation by 3Fax-peracetyl Neu5Ac (3Fax-PN, a sialyltransferase inhibitor) combining anti-PD-L1 treatment achieved a remarkable suppressive effect by reinvigorating the anti-tumor activity of T cells [80].

Targeting abnormal glycosylation, e.g., fucosylation, is also a potential therapeutic strategy in HCC. Pharmacological inhibition of FUT1-mediated α1,2-fucosylation by 2DGal was found to suppress HCC cell proliferation, self-renewal and tumor-initiating ability [68]. In addition, treatment of HCC with 2-fluoro-L-fucose (2FF), an analog of L-fucose, abolished core fucosylation of EGFR and integrin β1 on the cell surface via limiting GDP-fucose biosynthesis, leading to impaired pro-tumorigenic signalings and subsequent suppression of HCC cell proliferation and migration [55]. 6-alkynyl-fucose (6-Alk-Fuc) is a novel fucosylation inhibitor that could deplete cellular GDP-fucose via direct targeting of GDP-fucose-synthesizing enzyme FX [90]. Administration of 6-Alk-Fuc potently suppressed migration and invasion of HCC cells without affecting cell proliferation [90]. Therefore, targeting cellular fucosylation represents a promising strategy for HCC treatment.

## Conclusion and future directions

The advancement in the knowledge of cancer-associated glycobiology has provided novel insights into the driving forces behind tumor initiation and progression. Glycosylation abnormalities are commonly observed in HCC, which reflect the change of protein properties and functions, especially for cell-membrane and secretory glycoproteins, which mediate cancer cell interactions, extracellular communications and host immune response. Changes in protein glycosylation could not only drive pro-tumorigenic properties such as proliferation, metastasis, and stemness but also contribute to tumor immune tolerance and therapy resistance, suggesting that glycosylation abnormalities can potentially serve as diagnostic and prognostic biomarkers in HCC. Meanwhile, the change of glycosylation machinery in HCC is associated with the response of patients to chemotherapy, targeted therapy, as well as immunotherapy. Targeting aberrant glycosylation thus provides a novel and promising approach to fight against cancer therapeutic resistance. To this end, several preclinical studies have been performed using in vitro HCC cell lines, or in vivo mouse xenograft models. For example, 2DGal is a deoxy hexose that inhibits synthesis of glycoproteins and administration of 2DGal to HCC cells would increase the therapeutic efficacy of sorafenib [68]. Meanwhile, silencing of GALNT10, an important enzyme for the synthesis of mucin-type oligosaccharides, would enhance cell apoptosis in HCC cells treated with sorafenib or doxorubicin [23]. However, there remain several questions understudied. Firstly, a variety of factors such as host genetics and inflammation which exert profound effects on the change of protein glycosylation remain to be elucidated. Secondly, the administration of broad-spectrum glycosylation inhibitors could also affect the neighboring adjacent normal tissues, thus drug-related toxicity should be considered. Thirdly, more clinical studies should be conducted to assess the clinical implications of cancer glycomics given that humans and mice exhibit distinct glycomes. Moreover, N-glycosylation is well-known to affect immune cell differentiation and maturation. N-glycan chain on the T cell receptor (TCR) and major histocompatibility complex (MHC) are important for maintenance of stable immune synapses, which are required for T cell activation [91]. Clarifying the aberrant glycosylation patterns in immune cells such as T and B will improve our understanding of HCC pathogenesis. Nevertheless, these pieces of information are missing, and future studies are warranted.

In conclusion, dysregulated protein glycosylation in HCC brings us new opportunities for the identification of useful diagnostic and prognostic biomarkers and the development of glycan-based treatments for HCC patients.

## Data Availability

The data are available upon request.
